# Molecular Profile Sensitization to House Dust Mites as an Important Aspect for Predicting the Efficiency of Allergen Immunotherapy

**DOI:** 10.3389/fimmu.2022.848616

**Published:** 2022-03-22

**Authors:** Victoria V. Rodinkova, Serhii D. Yuriev, Mariia V. Kryvopustova, Vitalii B. Mokin, Yevhenii M. Kryzhanovskyi, Andrii I. Kurchenko

**Affiliations:** ^1^ Department of Pharmacy, National Pirogov Memorial Medical University, Vinnytsia, Ukraine; ^2^ Centre of Moleclar Allergology, Functional and Family Clinic “FxMed”, Kyiv, Ukraine; ^3^ Department of Clinical Immunology and Allergology, Bohomolets National Medical University, Kyiv, Ukraine; ^4^ Department of Pediatrics No2, Bohomolets National Medical University, Kyiv, Ukraine; ^5^ Department of System Analysis and Information Technologies, Vinnytsia National Technical University, Vinnytsia, Ukraine

**Keywords:** house dust mites’ sensitization, molecular allergens, efficiency of allergen immunotherapy, HDM, AIT, regional peculiarities of HDM sensitization

## Abstract

House dust mite (HDM) allergens are considered to be one of the most common causes of asthma and allergic rhinitis in the world. Cysteine proteases Der p 1 and Der f 1 (group 1) and also NPC 2 family proteins Der p 2 and Der f 2 (group 2) of *D. pteronyssinus* and *D. farinae* respectively are considered the main allergens of HDMs. The difference in the sensitivity of the population to these and other allergy causing components of HDM determines the treatment strategy. Thus, the purpose of this work was to determine the pattern of sensitization of the Ukrainian population to individual allergy causing molecular components of HDM in order to improve treatment strategies for the HDM allergy in various regions of Ukraine. To determine the molecular profile of sensitization to HDM, the data of multiplex allergy test Alex2 have been obtained from 10,651 patients. The sample included 57.86% children under the age of 18 and 42.14% adults. A Python language-based statistical analysis was performed, in order to group patients by sensitization to individual molecules and their combinations, regarding the age and geographical location of the patients. Simultaneous sensitization to Der f 2 and Der p 2 allergens was the most common among the entire group Simultaneous sensitization to 5 molecules—of group 1 (Der p 1 and Der f 1), group 2 (Der f 2 and Der p 2), and Der p 23—was the second most common for entire dataset and for the children group. This pattern differed in adults, where monosensitization to Der p 23 occupied the second position, suggesting that this molecule is an important factor of HDM allergy in Ukraine. Of the 16 analyzed regions, sensitization to Der p 23 prevailed in 2 Western regions of Ukraine. In the rest of the regions combination of Der p 2 and Der f 2 was the most prevalent. The established character of population sensitization to HDM in Ukraine is a good prognostic marker of allergen immunotherapy (AIT) efficacy.

## Introduction

House dust mite (HDM) allergens are considered to be the most common triggers of asthma and allergic rhinitis in the world. Due to their ability to provoke allergic responses, type 1 hypersensitivity in particular, the HDMs are unique representatives of the Arachnida class. Most of the Arachnids are plant pathogens and also plant and animal parasites (1). However, it is believed that only 1–2% of the world population are sensitive to mite allergens. Although at the first glance this figure seems to be insignificant, the allergic sensitivity to the HDM greatly varies from country to country. Moreover, in the developed countries, the HDM allergic sensitivity index is significantly higher. For instance, in the countries of Europe it exceeds 20% and in some communities of North America it reaches 40%. As for the children of Taiwan that have type I allergy, the sensitivity to dust mites is over 80% (2).

Moreover, some research results claim that at least 50% of the bronchial asthma patients and 45% of the allergic rhinitis patients are sensitized to the HDMs (3).

Therefore, the occurrence of sensitization to HDMs and their effect on the humans depend on various environmental factors, namely, the climate and microclimate, microhabitats of mites in domestic environment, etc.

Nowadays, there are several species of the HDM, which are known to cause allergic sensitization.

According to http://allergen.org/, the official website of the allergens systematic classification, approved by the WHO and the Allergen Nomenclature Sub-committee of the International Union of Immunological Societies (WHO/IUIS), most allergenic proteins, 31 and 36 respectively, are found in *Dermatophagoides pteronyssinus* or the European HDM and *Dermatophagoides farinae*, the American HDM (4). Both mites contain basic allergens—group 1 and 2 proteins. The two species are highly homologous, with widely spread cross reactions (5).

The allergens of two other dust mite species have been described as well: *Dermatophagoides microceras* (1 allergenic protein Der m 1) and *Euroglyphus maynei*, with 5 allergens, from Eur m 1 to Eur m 4 and Eur m 14 (4).

The main allergenic proteins of HDMs correspond to several classes: cysteine proteases (Der f 1, Der p 1, Der m 1, Eur m 1)—group 1, NPC2 family (Der f 2, Der p 2, Der m 2, Eur m 1)—group 2. Der p 10 and Der f 10 are the tropomyosins, Der p 15 and Der f 15—chitinase-like proteins and chitinases respectively, Der p 20 and Der f 20 refer to the arginine-kinases.

Of the allergens mentioned above, cysteine proteases Der p 1 and Der f 1 of, *D*, *pteronyssinus* and *D. farinae* (6) respectively are considered to be the main ones. More than 80% of people allergic to HDMs are sensitive to those (7).

However, another protein, Der p 23, which is also called a major component of the HDM, has recently gained considerable clinical significance (8). This allergen is a peritrophin-like protein domain (PF01607). Der f 23 is also a peritrophin-like protein and Der f 37 is the protein with the peritrophin-A domain (4).

Other clinically significant HDM allergens are Der p 5, Der p 7, and Der p 21. What is more, according to the scientific literature, Der p 7 has the most expressed allergenic properties of all the allergens in the list (9). However, the data may be relevant only for the patients who participated in the clinical trial, as the pattern of sensitization is affected by several factors, namely, age of patients, the geographical region, poly- and mono-sensitization and also sensitization to certain allergen sources (10).

Thus, the difference in sensitivity of the population to these and other allergic components of HDM defines clinical course of an allergic disease and its treatment regimen. Although it is often possible to control the respiratory allergy to HDM with symptomatic drugs, satisfactory disease control in some patients is unachievable (10).

This fact predisposes the decision to choose allergen immunotherapy as the treatment method for the HDM allergic hypersensitivity. In its turn, being based on the results of allergy molecular diagnostics, the AIT allows to select candidates for treatment and predict the success of the allergen-specific immunotherapy (AIT) depending on the protein allergen the patient is sensitized to.

Thus, it has been proven today, HDM allergens are an important factor in allergic rhinitis and bronchial asthma development. However, some issues concerning clinical relevance of certain allergens to allergy development and to the choice of therapy still remain undefined (2).

Considering all of the above-mentioned facts, which confirm that certain therapeutic approaches still do not take into consideration the sensitization profiles of patients (11), the purpose of the study was to assess sensitization of the population of Ukraine to certain allergenic components of HDM in order to improve treatment regimens with the help of AIT for patients in various regions of Ukraine.

## Materials and Methods

In order to determine the HDM sensitization profile, data from 16,309 people who live in 16 different regions of Ukraine and who had taken the Alex2 molecular test were collected.

Inclusion criteria for the study were following: allergic disease in the anamnesis (allergic rhinitis, atopic dermatitis, bronchial asthma solely or in combination). Patients were selected based on the analysis of their medical history, which was carried out in clinics in Ukraine, where allergists examined the patients.

All patients signed informed consent before testing. Among others, it included paragraph about possible usage of impersonalized data of patients for scientific purposes. All studies were approved by Ethics Committees, which are functioning at every clinic, which obtained the results.

sIgE levels to Der f 1, Der f 2, Der p 1, Der p 2, Der p 5, Der p 7, Der p 10, Der p 11, Der p 20, Der p 21 and Der p 23 allergens were components of the ALEX test used for analyses. The studied patients lived in various biomes, namely, the Forest, Forest Steppe and Steppe zones. Inhabitants of 16 out of 25 administrative units of Ukraine were involved into the study.

Most of the tested patients were from the following cities: 19.62%—Kyiv (Forest zone), 14.48%—Odessa, 7.7%—Kharkiv (Steppe zone), 7.55%—Dnipro (Forest-Steppe zone).

After the primary analysis of the obtained database by the geographical distribution and age composition, the data of 10,651 people were included in the further study, as their age and geographical origin could be clearly defined. This group included 6,163 (57.86%) children under the age of 18 and 4,488 adults (42.14%).

In order to automate the data processing, a set of applications was developed using the Python-language of programming.

All patients were grouped by regions, in order to simplify detection of geographical similarities. In order to detect sensitization consistencies, the data were transformed into a binary system. 0.35 kU/L was determined as the threshold sensitization to HDM allergens. The sensitization that equalled or exceeded 0.35 was marked as 1, lower values—as 0.

While analyzing the sensitization of the population of Ukraine to various molecules of Der p and Der f (of each group separately and of two groups simultaneously), three criteria were considered: J_1_ = «Age», J_2_ = «Geographic distribution» and the combined criterion – J_12_ = «Geographic distribution of various age groups».

In criterion J_1_ «Age», the authors analyzed the data of all patients with sensitization to one or several molecules of Der p and Der f, namely, “Der f 1–Der f 2–Der p 1–Der p 2–Der p 5–Der p 7–Der p 10–Der p 11–Der p 20–Der p 21–Der p 23”. The prioritized variants such of molecular complexes were calculated for different age groups (for children and adults).

In order to study criterion J_2_, «Geographic distribution», the number of patients with sensitivity to each molecule from Der p and Der f group was calculated by region. This means that sensitization to Der f 1, Der f 2, Der p 1, Der p 2, Der p 5, Der p 7, Der p 10, Der p 11, Der p 20, Der p 21, and Der p 23 was calculated separately. Then, according to the number of sensitized patients, the authors determined prevailing molecules for regions, finding the first and the following maximums, i.e., molecules that the patients were sensitized most often to. If the incidence of the first (absolute) maximum exceeded the next incidence by more than 5%, than only the molecule with the highest incidence was determined as the main sensitizing molecule in the region. If the sensitization incidence to the first maximum equalled to the sensitization of the second maximum, or differed from it by less than 5%, it was concluded that the citizens are sensitized to both molecules simultaneously.

In order to study criterion J_12_, «Geographical distribution of various age categories», the authors used the algorithm of calculating criterion J_2_ = «Geographical distribution». However, it was not applied to the whole dataset at once, but for children and for adults separately. The data were simultaneously analysed for Der p and Der f molecules.

And then we determined 1–2 priority molecules for the region, based on the number of patients sensitizing to them, according to the following algorithm: a number of patients sensitizing to a certain molecule were calculated and then divided by the total number of patients in this region who sensitize to at least one of these molecules. The first (absolute) maximum of this series was determined and the molecule that corresponds to it. Then, for the final value, the null hypothesis was tested that the second maximum (the second largest value) can be ignored, because only the absolute maximum reflects the main sensitization of the region. Since the series is short (up to 11 values—according to the number of molecules, to which sensitization is theoretically possible in the region), the Student’s distribution method was used. For each value, the p-value was calculated using the functions of the Python library SciPy: stats.ttest_1samp with the parameter alternative = ‘greater’, as it was important to analyze the right tail of the histogram, i.e., to know whether there is another maximum. For most molecules, the hypothesis was considered unconfirmed for 0.002 level. In the unclear cases, the value of the series was taken for which the p-value was the smallest and differed by no more than 5% from the p-value for the absolute maximum, i.e., the one that was sufficiently similar (often identical) to it, so that it could be assumed that the null hypothesis regarding it was not confirmed.

## Results

Sensitization to at least one HDM allergens has been established in 2,875 people, which made up 27.00% of the studied sample. The number of sensitized children was 2.26 times greater than that of the adults: 1,993 (69.32%) children vs 882 (30.68%) adults.

**Figure 1:**
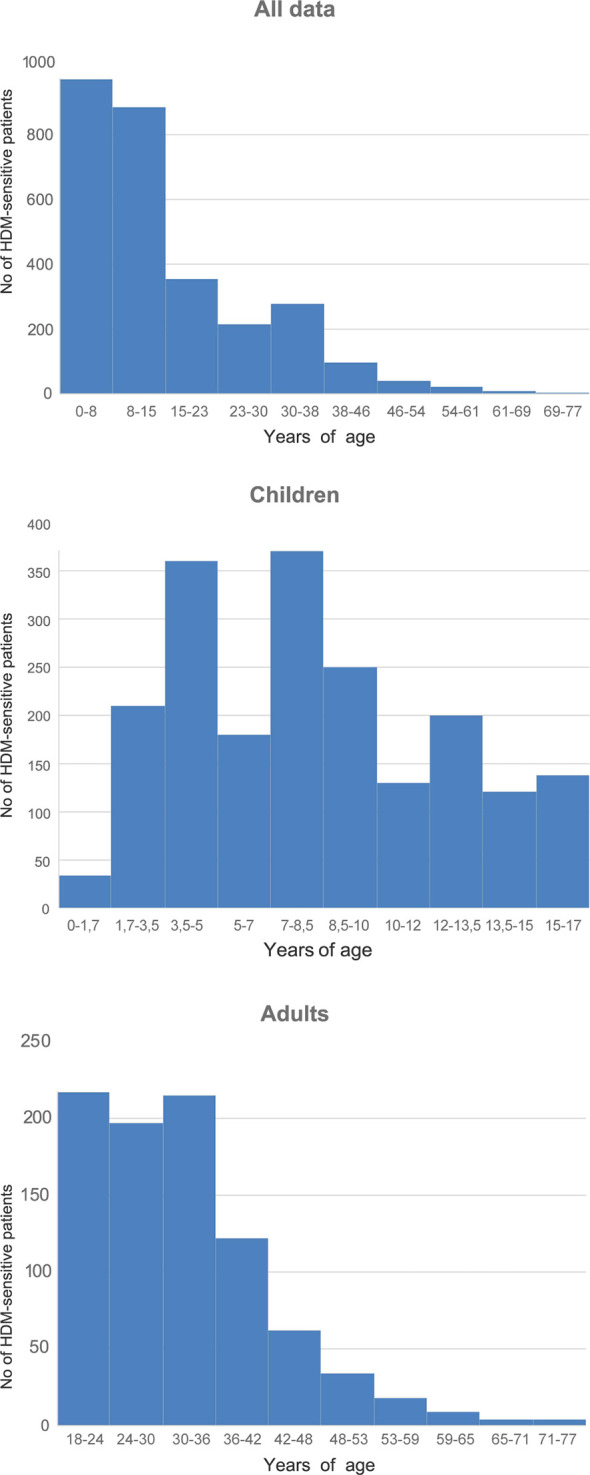
Range of the studied subjects by age. Mean tIgE of sensitized group was 332.82 ± 516.55.

Mean age of the HDM-sensitized group was 19.56 ± 16.65 (M ± σ). Children were 7. 29 ± 4.29 years in average, adults—36.41 ± 11.9. Group of children of 0–8 y.o. was the most numerous in the entire studied group. Children of 3.5–5 and 7–8.5 years dominated in the children group. In adults, people of 18–24 and 30–36 y.o. predominated (**Figure 1**).

Sensitization to allergen Der f 2 prevailed in the profiles of diagnosed patients. It was observed either separately, or in combination with other allergenic molecules, in 2,106 (73.25%) patients sensitive to the HDM allergens. Sensitivity to allergen Der р 2—either mono-sensitivity, or combined sensitivity to other allergens—was observed in practically the same number of patients, namely, 2,073 (72.10%).

The third most prevalent was sensitization to allergen Der р 23, observed in 1,602 (55.72%) patients. The sensitization to Der р 1 was detected in 1,567 (54.50%) patients, and 1,553 (54.01%) patients demonstrated sensitization to Der f 1.

Sensitization to allergen Der р 21 was found in 786 (27.34%) patients, to Der p 5 in 818 (28.45%) and to Der р 7 in 649 (22.57%) patients.

The lowest sensitization levels were registered for the following allergens: Der р 20–234 patients (8.14%), Der р 10–177 (6.16%), Der р 11–16 patients (0.56%).

The highest sensitivity in children and in adults alone was also recorded to Der f 2 and Der p 2 allergens.

Mean sIgE of the entire group was clearly higher to both Der f 2 and Der p 2 (27.43 ± 17.26 kU/L and 24.65 ± 17.19 kU/L accordingly). The same tendency was seen for the children and adults separately. Mean sensitization to Der f 2 Der p 2 in the group of children was 30.34 ± 16.05 kU/L and 29.18 ± 16.13 kU/L correspondingly. Mean level of sIgE to Der f 2 in adults was 26.57 ± 17.28 kU/l, to Der p 2 it was 25.97 ± 17.26 kU/L. These values were the highest in data series of mean sIgE-s for both children and adults. Der p 21 was the third by the mean sIgE for general cohort and for children and adults too (19.7 ± 17.28 kU/L, 22.10 ± 16.75 kU/L and 17.95 ± 16.89 kU/L respectively). Sensitization to the Der p 11 was the lowest one and constituted 1.65 ± 3.34 kU/L for entire cohort and 1.28 ± 1.44 kU/L for children and 1.91 ± 4.19 kU/L for adults. Der p 20 was the last but one with following values: 9.49 ± 12.21 kU/L for general cohort, 11.97 ± 14.02 kU/L—for children and 9.57 ± 12.29 kU/L—for adults. The rest values of mean sIgE varied from 14.33 ± 16.26 kU/L (Der p 10 in adults) to 19.70 ± 14.98 kU/L (Der p 7 in children) (**Table 1**).

**Table 1:** Share of patients sensitized to molecular allergens of HDMs.

Molecular allergens	Mean sIgE, kU/L M±σ	No. of patients sensitized to corresponding allergen	% of all patients sensitized to corresponding allergen	Mean sIgE in the group of children, kU/L M±σ	No. of children sensitized to corresponding allergen	% of children sensitized to corresponding allergen	Mean sIgE in the group of adults, kU/L M±σ	No. of adults sensitized to corresponding allergen	% of adults sensitized to corresponding allergen
**Der f 2**	27.43 ± 17.26	2,106	73.25	30.34 ± 16.05	1,474	73.96	26.57 ± 17.28	632	71.66
**Der p 2**	24.65 ± 17.19	2,073	72.1	29.18 ± 16.13	1,461	73.31	25.97 ± 17.26	612	69.39
**Der p 23**	16.06 ± 15.24	1,602	55.72	16.67 ± 14.48	1,161	58.25	16.03 ± 15.19	441	50
**Der p 1**	15.21 ± 13.23	1,567	54.5	16.96 ± 13.43	1,146	57.5	14.97 ± 13.36	421	47.73
**Der f 1**	15.02 ± 14.01	1,553	54.02	15.77 ± 13.47	1,167	58.55	14.67 ± 14.00	386	43.76
**Der p 5**	14.06 ± 14.34	818	28.45	14.56 ± 14.36	613	30.76	13.90 ± 14.02	205	23.24
**Der p 21**	19.7 ± 17.28	786	27.34	22.10 ± 16.75	535	26.84	17.95 ± 16.89	251	28.46
**Der p 7**	16.68 ± 6.89	649	22.57	19.70 ± 14.98	472	23.68	16.57 ± 15.59	177	20.07
**Der p 20**	9.49 ± 12.21	234	8.14	11.97 ± 14.02	129	6.47	9.57 ± 12.29	105	11.9
**Der p 10**	13.23 ± 13.25	177	6.16	12.43 ± 15.36	121	6.07	14.33 ± 16.26	56	6.35
**Der p 11**	1.65 ± 3.34	16	0.56	1.28 ± 1.44	6	0.3	1.91 ± 4.19	10	1.13
**Total sensitized to HDMs**		2,875			1,993			882	

In relation to the aggregation of allergens, we got 189 distinct profiles in 2,875 subjects. Among them co-sensitization to Der f 2 and Der p 2 was prevalent in both age groups. It was detected in 12.52% of the patients. This sensitization was also most common in each separate age group. Among the children, the occurrence of co-sensitization to Der f 2 and Der p 2 was 11.74%, and among the adults it was 14.29%.

The next most prevalent sensitization type characteristic of all the population of Ukraine was simultaneous sensitivity to 5 molecules—allergens of groups 1 and 2 (Der f 1, Der p 1, Der f 2, Der p 2) and to peritrophin-like protein Der p 23. It was registered in 9.22% of the studied patients. The third most prevalent in all age groups was simultaneous sensitization to all major molecular components of HDMs—Der f 1, Der f 2, Der p 1, Der p 2, Der p 21, Der p 23, Der p 5, and Der p 7, and it was registered in 7.72% patients.

**Figure 2:**
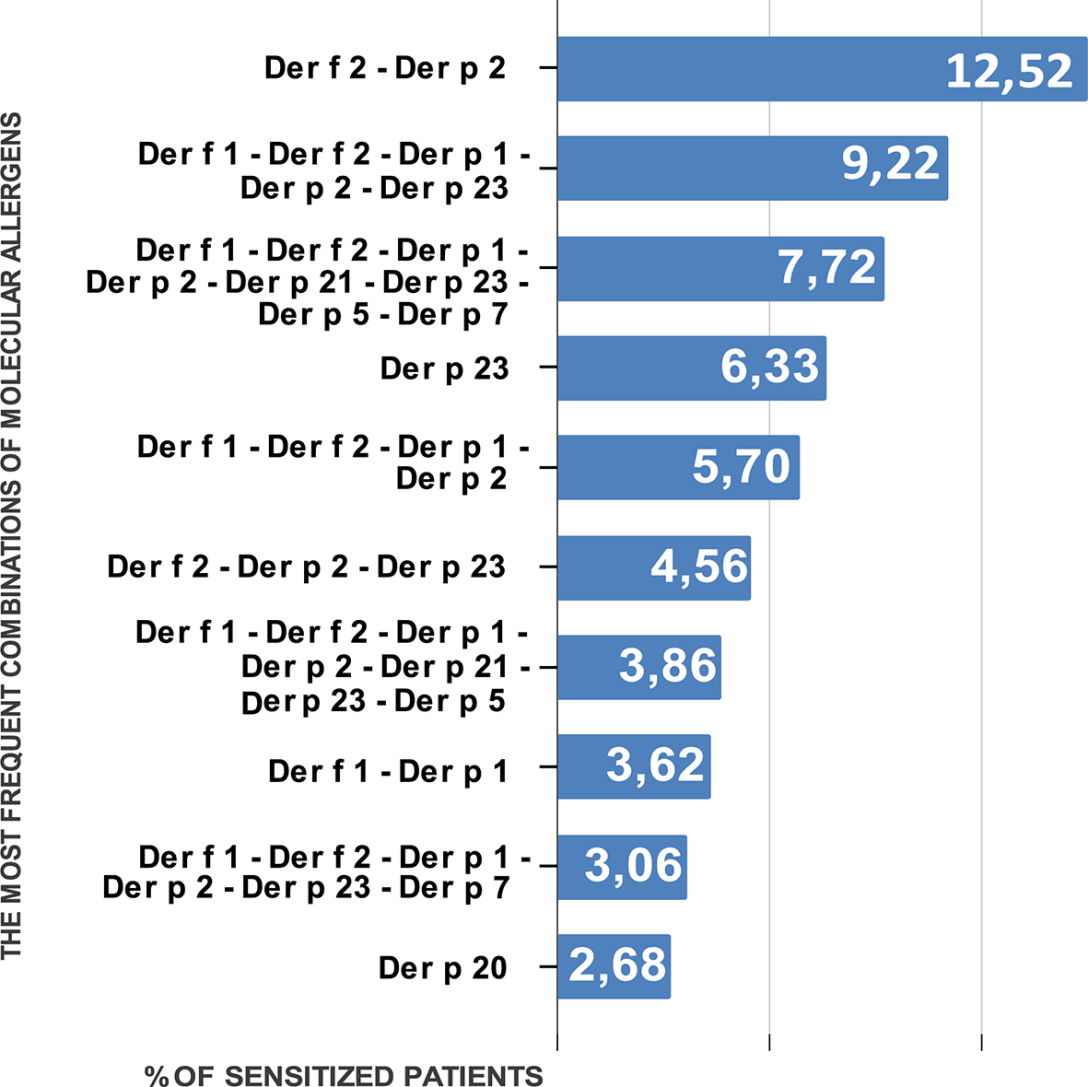
Percentage of people sensitized to different combinations of molecular allergens of HDMs.

Next in line was monosensitization to Der p 23. It was registered in 6.33% of the patients. This was the most common monosensitization among patients of all age groups. Sensitization to Der p 20 was the next most prevalent monosensitization. However, it was (**Figure 2**) only the tenth in the overall list of sensitization registration (2.68% of patients).

Monosensitization to each allergen from groups 1 and 2 was registered in 0.14% (Der p 2)–1.3% (Der f 1) of patients.

The nature of sensitization in the children group was similar to that observed in the general group. The first three positions were the same; the fourth most prevalent was sensitization to allergens of groups 1 and 2: Der f 1, Der f 2, Der p 1, and Der p 2. It was registered in 5.82% of the patients. Monosensitization to Der p 23 was registered in 5.37% of cases. Monosensitization to Der f 1 and Der p 1 was observed in 1.51 and 0.55 % of patients, respectively.

Among the adults, the second by incidence, after simultaneous sensitization to Der f 2 and Der p 2, was monosensitization to Der p 23. It was registered in 8.5% of patients. Simultaneous sensitization to group 1 and 2 allergens and to Der p 23 was observed in 7.48% of the patients aged over 18 years.

The next by frequency of monosensitization to the major allergens was sensitivity to Der f 2 (2.04%), which occurred as often as simultaneous sensitivity to Der f 1 and Der p 1. Monosensitization to these allergens occurred in 0.34% (Der p 1) and 0.91% (Der f 1) of patients.

In terms regional variations, simultaneous sensitization to Der f 2 and Der p 2 was the most common. It was registered in 14 out of 16 regions. In two regions of Western Ukraine (Zakarpattia and Khmelnytskyi regions) sensitization to Der p 23 was most prevalent.

Patterns of the sensitization were similar in children group. Simultaneous sensitization to Der f 2 and Der p 2 prevailed in 12 regions; sensitization to Der p 23 prevailed in Zakarpattia and Khmelnytskyi regions. No children sensitized to HDMs were registered in the Mykolayiv region (South) and Der f 2 was the leading allergen in the Kherson region (South).

In the adult group, sensitization to Der f 2 was most prevalent in the Dnipropetrovsk (Centre) and Zakarpattia (West) regions. Sensitization to Der p 23 was most often registered in the Poltava (East) and Kherson (South) regions. Among adults of the Zhytomyr, Khmelnytskyi and Rivne (West) regions sensitization to HDMs was not registered. The Cherkasy region (Centre) was excluded from the analyses too as only two adult patients with sensitisation to HDM were detected there.

**Figure 3:**
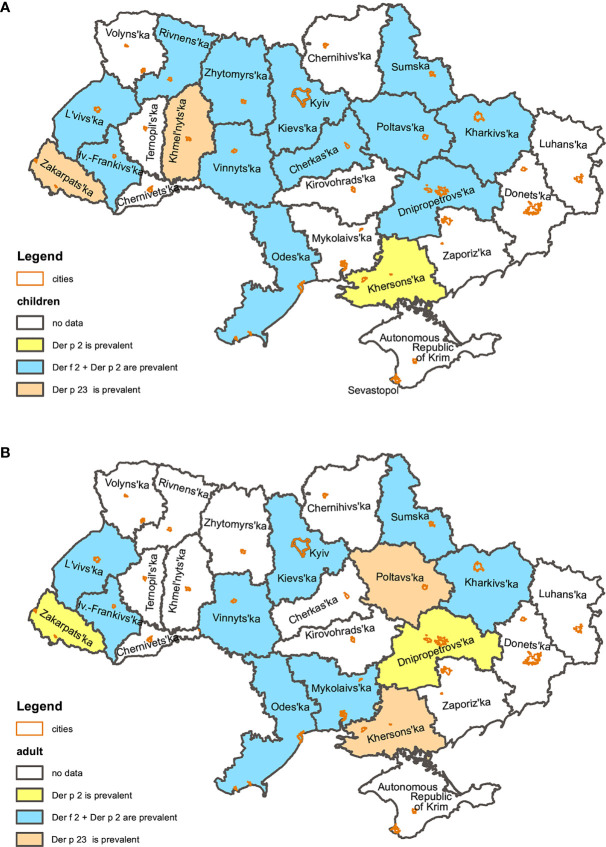
Regional patterns of children **(A)** and adult **(B)** sensitization to HDMs in Ukraine.

In the rest of the regions simultaneous hypersensitivity to Der f 2 and Der p 2 (**Figure 3**) was most prevalent.

## Discussion

The study results showed that in Ukraine the HDM allergen sensitization was more common in children than in adults. This may correlate with the understanding that HDM sensitization develops since early childhood and plays an important role in the development of the so-called “Allergic March” (12).

The analysis has shown that sensitization to group 2 allergens—Der f 2 and Der p 2—was most prevalent both among the population of Ukraine in general and among children and adult groups separately. The second most prevalent was polysensitization to group 1 and group 2 allergens and to Der p 23.

The study detected the regions of Ukraine with the most prevailing sensitization to allergen Der p 23. This once again confirms the importance of assessing sensitization to this very molecule as a yet another major HDM component, which is of high therapeutic significance.

The data on sensitization to Der p 1, Der p 2 and Der p 23 allergens are clinically significant as early sensitization to these allergens is related to the bronchial asthma development (13).

It is considered that patients with asthma are sensitive to a wider range of HDM allergens than those who do not suffer from asthma. Sensitization to Der p 1 and Der p 23, developing before the age of five, is an important predictor of bronchial asthma development at a school age (14).

The clinical role of Der p 5 and Der p 7 allergens has not been completely studied yet. Our data evidenced the relatively small spread of sensitization to them among the Ukrainian population. The sensitization to them was the most frequent among people, simultaneously polysensitized to all major HDM allergens of groups 1, 2, Der p 21 and Der p 23. Monosensitization to Der p 5 and Der p 7 was observed in less than 1% of examined population of Ukraine.

Sensitization to Der p 7 can also be an asthmatic marker. As Curin et al. state, patients with IgE antibodies to Der p 7 reported breathing difficulties more often and were more prone to asthma development, even though a mild one, than those patients who were not sensitized to Der p 7 (9).

There is also evidence that patients with allergic rhinitis respond to a bigger number of HDM allergens and are more often sensitized to Der p 23 and Der p 7 (3).

Thus, the pattern of sensitization of the patient to HDM allergens may potentially indicate the peculiarities of the clinical course, which should be expected in a particular patient.

The peculiarities of a sensitization profile of a patient may serve as a predictor of the efficacy of HDM allergy treatment. For AIT is the only treatment method, which modifies the disease manifestations. Both Subcutaneous Immunotherapy (SCIT) and Sublingual Immunotherapy (SLIT) with HDM extracts have shown safety and efficacy in decreasing allergy symptoms and also in improving quality of life of patients with allergic rhinitis and asthma. The AIT also shows the long-term remission effect after the completion of the treatment and prevents new sensitizations (15). The choice of АІТ in patients sensitized to various HDM allergens and prediction of its efficacy may be made based molecular sensitization data. According to Curin et al. (16), the key limiting factor for the HDM allergen-based vaccines used in the therapeutic practice nowadays is the fact that natural HDM extracts contain insufficient quantity of such important allergens as Der p 5 and Der p 7 that are an immunogenic component needed for triggering appropriate immune response. Thus, allergen extract based vaccines may be less effective in patients, sensitive to these allergens. There is a hypothesis that predominantly Der p 2 and Der p 1 are immunogenic allergens, while the other allergenic molecules in the extract demonstrated low immunogenicity and, thus, they did not induce the IgG-response, which is the key for tolerance development. Rodríguez-Domínguez et al. studied AIT efficacy in patients with various sensitization profiles and established that АІТ induced protective IgG, mainly to Der p 1 and Der p 2, and less often to Der p 23, but not to other important allergens, such as Der p 5, Der p 7 and Der p 21 (17). This confirms higher clinical efficacy of AIT in patients, sensitized only to Der p 1 and/or Der p 2 in comparison with patients sensitized to other allergens.

On the other hand, Stranzl et al. (18) showed that the sublingually introduced allergens contain immunologically significant quantities of all three main HDM allergens for induction of a Der p 23-specific IgG4 in the course of HDM SLIT. Also, these authors concluded that stratification based on sensitization of patients to Der p 23 is clinically insignificant before the start of AIT.

And as we can see, the most prevalent patients in the Ukrainian population are those sensitive to group 2 allergens and to Der p 23 and patients with sensitization to Der p 5, 7, 20, 21 are quite rare. This proves that AIT is recommended to most patients with clinical manifestations of HDM allergy.

Therefore, the selection of patients with HDM allergy according to their molecular sensitization profiles may increase the efficacy of AIT. However, the treatment regimen should different for various groups of population, and, in the end, individual treatment regimens should be developed.

New clinical trials of clinical relevance of certain HDM allergens are needed and the AIT optimizing strategies should be developed in order to increase its efficacy in patients with sensitization to Der p 5, Der p 7, and Der p 21, which is also suggested by other authors (9).

Thus, children in Ukraine were 2.26 times more often sensitized to the HDM allergens than adults.

The key sensitizing HDM molecules in our geographical area are Der p 2 and Der f 2.

The second most prevalent among the adults is monosensitization to Der p 23, and among children—polysensitization to group 1 and 2 allergens and to Der p 23. Such polysensitization in the third most prevalent in the adult group.

Sensitization to Der p 5, Der р 21, and Der р 7 allergens was registered in 28.45–22.57% patients, allergic to HDMs. Even though sensitization to Der р 20 and Der р 10 allergens was less expressed, it was also observed a considerable share of the studied patients—8.14 and 6.16% respectively. Thus, sensitivity to Der p 5, Der p 7, Der p 10, Der p 20, Der p 21, and Der p 23 allergens should be considered in the course of selecting treatment strategies for patients with HDM allergy.

In general, the established character of population sensitization to HDM in Ukraine is a good prognostic marker of AIT efficacy, even though currently existing extract are rather mainly aimed at developing tolerance to group 1 and group 2 allergens, and less so to Der p 23.

The study has established different sensitization character in different age groups (adults and children) and in different geographical regions of Ukraine. The defined tendencies should be taken into consideration while selecting the AIT strategy and predicting its efficacy both regionally and individually.

## Data Availability Statement

The original contributions presented in the study are included in the article/supplementary material. Further inquiries can be directed to the corresponding author.

## Author Contributions

Designed the project: VR, MK, and SY. Supervised the project: AK and SY. Collected data: SY, MK, and AK. Analyzed data: VM and YK. Provided advice: SY, MK, VM, and AK. Wrote the manuscript: VR, MK, VM, and YK. All authors listed have made a substantial, direct, and intellectual contribution to the work and approved it for publication.

## Conflict of Interest

The authors declare that the research was conducted in the absence of any commercial or financial relationships that could be construed as a potential conflict of interest.

## Publisher’s Note

All claims expressed in this article are solely those of the authors and do not necessarily represent those of their affiliated organizations, or those of the publisher, the editors and the reviewers. Any product that may be evaluated in this article, or claim that may be made by its manufacturer, is not guaranteed or endorsed by the publisher.
